# Supplementary material for: Antioxidative and Mitochondrial Protection in Retinal Pigment Epithelium: New Light Source in Action
Source: Int J Mol Sci. 2023 Mar 1;24(5):4794. doi: 10.3390/ijms24054794 (PMC10003667; doi:10.3390/ijms24054794)
Supplement: Supplementary file 1 [file ijms-24-04794-s001.zip › ijms-2099546-supplementary.pdf]

Supplementary material for

Anti-oxidative and Mitochondrial Protection in Retinal Pigment Epithelium: New Light Sourcein Action

Ming Jin †, Xiao-Yu Zhang †, Qian Ying, Hai-Jian Hu, Xin-Ting Feng, Zhen Peng, Yu-Lian Pang, Feng Yan and Xu Zhang *

Correspondence: Xu Zhang
E-mail: ndfsyk092090@ncu.edu.cn

This PDF file includes: Table S1 and Figure S1-S5

Table S1. Primary antibodies covered in supplementary material.

Antibody	source	Catolog.No	Type of Ab	Dilution	MW
SIRT1	CST	9475s	Rabbit mAb	1:1000	120
Ki67	abcam	Ab15580	Rabbit pAb	1:100	\

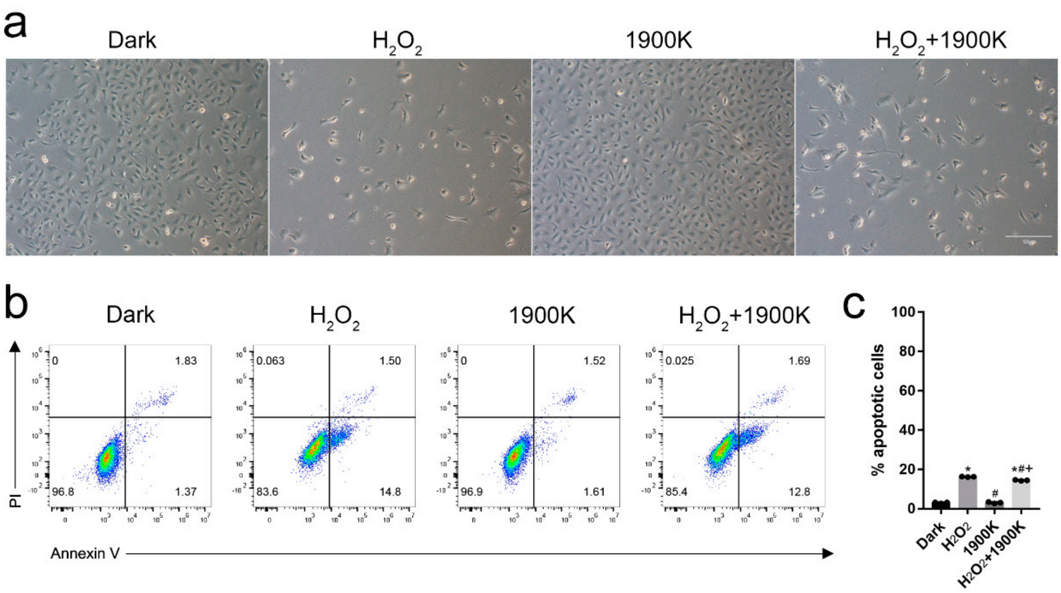

Figure S1. The 1900 K light-emitting diodes (LEDs) could not protect ARPE-19 cells in the light post-treatment paradigm. (**a**) The morphology of cells in bright field. We observed that the state of cells in the Dark and 1900 K LEDs groups looked similar. The cells in the hydrogen peroxide (H_2O_2) group and the H_2O_2+1900 K group were in poor condition--the cell connections between cells had been destroyed accompany with a layer of dead cells floating. What's more, the number of cells in H_2O_2 group and H_2O_2+1900 K group was much less than Dark group and 1900 K group. (**b**) Cell death of four groups. (**c**) Quantitative analysis of apoptosis rate. Asterisk (*): Significantly different from Dark (one-way ANOVA, H_2O_2 & H_2O_2 +1900 K: $p < 0.0001$). Hash sign (#): Significantly different from H_2O_2 (one-way ANOVA, 1900 K: $p < 0.0001$, H_2O_2 +1900 K: p=0.0002). Plus sign (+): Significantly different from 1900 K (one-way ANOVA, $p < 0.0001$). Flow cytometry results showed the apoptosis rate of the dark and 1900 K group was the same, and the apoptosis rate of H_2O_2 group was slightly higher than that of H_2O_2+1900 K group. More cells died in H_2O_2 group and H_2O_2+1900 K group than in dark and 1900 K group. The data are presented as the mean ± SD, n = 3. Bars in the bright field, 50 μm.

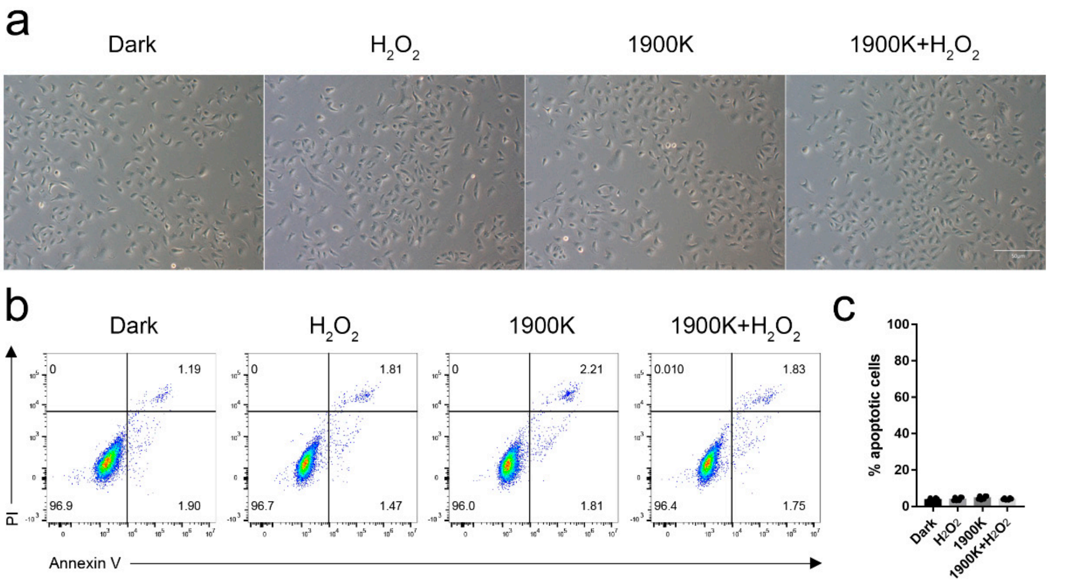

Figure S2. The morphology and cell apoptosis of ARPE-19 cells kept no changes in the light pre-treatment paradigm. (**a**) The morphology of cells in bright field. There was no difference in the morphology and number of cells among four groups. (**b**) Cell death of four groups. (c) Quantitative analysis of apoptosis rate. Apoptosis rates of the four groups tended to be the same. The data are presented as the mean ± SD, n = 6. Bars in the bright field, 50 μm.

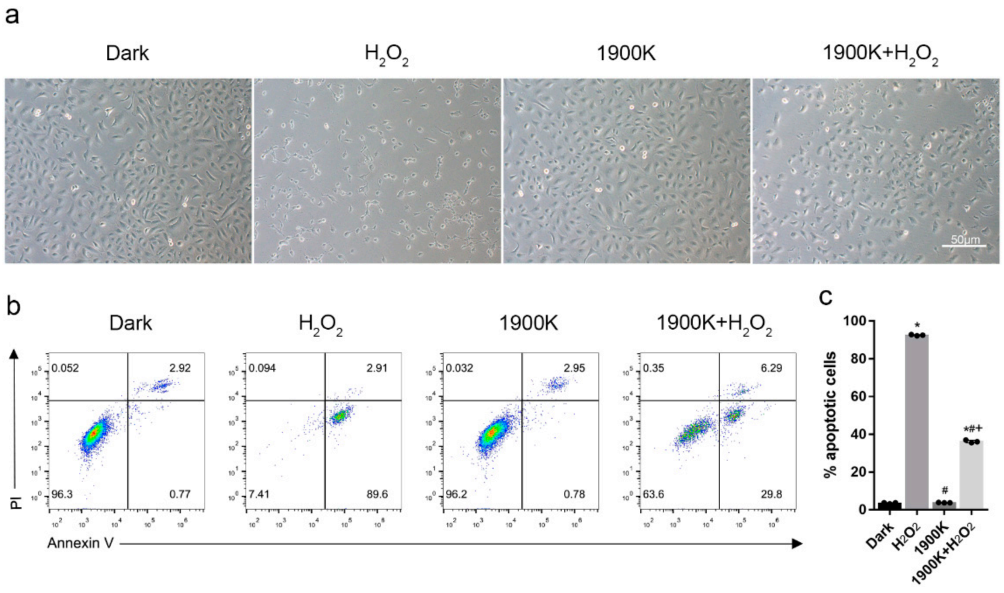

Figure S3. 1900 K LEDs pre-illumination could resist the damage of 1000 μM H_2O_2 for 24 h. (**a**) The morphology of cells in bright field. The state of cells in the Dark and 1900 K LEDs groups looked similar. However, the number of cells in the H_2O_2 group was less than that in the 1900

K+H_2O_2 group, and the state of cells in the 1900 K+H_2O_2 group was better than that in the H_2O_2 group. (**b**) Cell death of four groups. (c) Quantitative analysis of apoptosis rate. Asterisk (*): Significantly different from Dark (one-way ANOVA, $p < 0.0001$). Hash sign (#): Significantly different from H_2O_2 (one-way ANOVA, $p < 0.0001$). Plus sign (+): Significantly different from 1900 K (one-way ANOVA, $p < 0.0001$). We observed that the apoptosis rate of the dark and 1900 K group was similar and lower than that of the 1900 K+H_2O_2 group. The apoptosis rate of the H_2O_2 group was significantly higher than that in the 1900 K+H_2O_2 group. The data are presented as the mean ± SD, n = 3. Bars in the bright field, 50 μm.

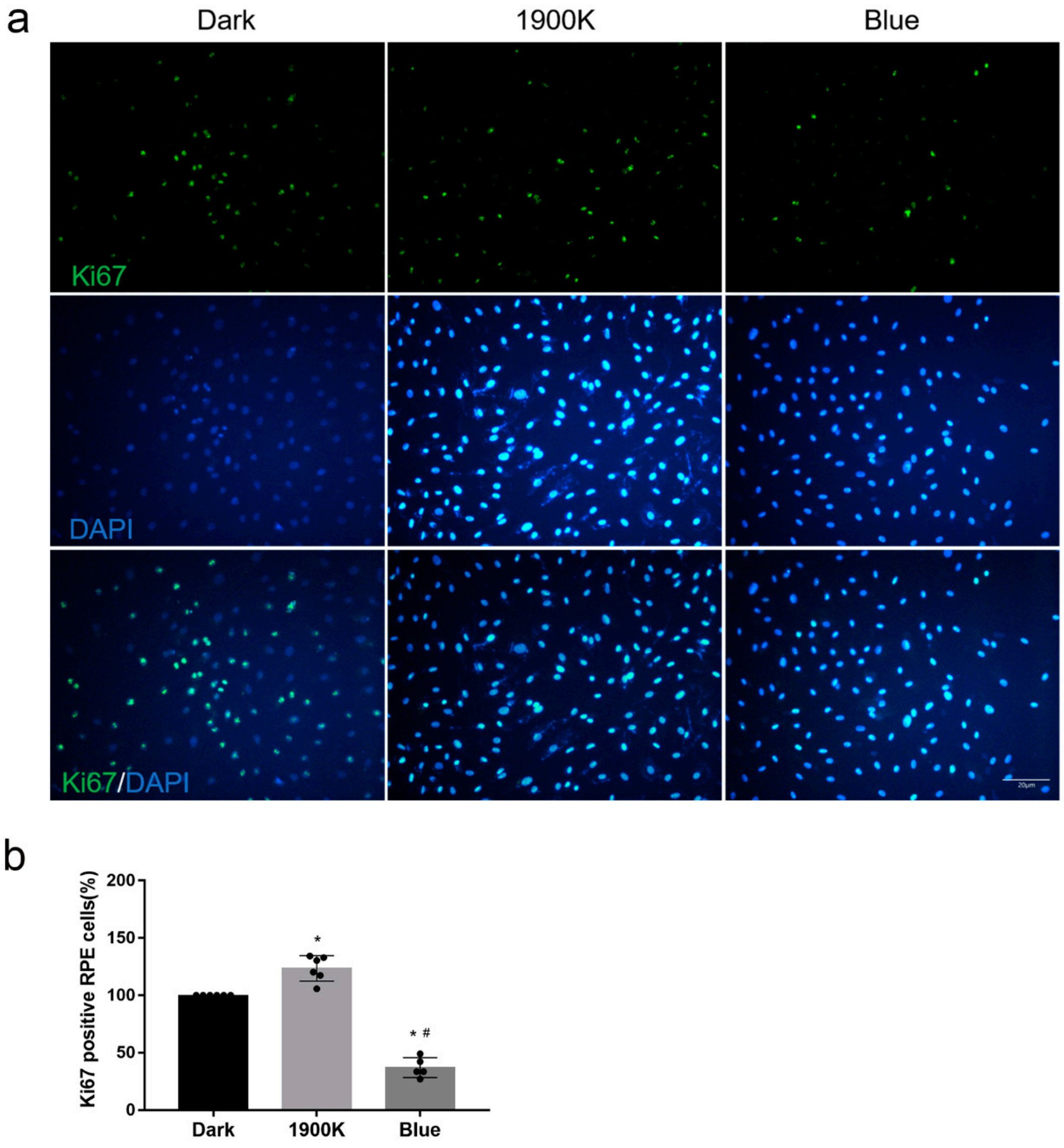

Figure S4. 1900 K LEDs could promote the proliferation of ARPE-19 cells. We illuminated ARPE-19 cells with 1900K LED and blue light 10w/m^2 for 48h, and found that the proportion of Ki67 positive cells in the 1900K group was the highest, while the proportion of Ki67 positive cells in the blue light group was the lowest. (A) The fluorescence results of Ki67 staining in three groups. (B) Quantitative analysis of Ki67 positive cells. n = 6, 6, 5. Asterisk (*): Significantly different from Dark

(one-way ANOVA, 1900 K: $p = 0.0005$, Blue: $p < 0.0001$). Hash sign (#): Significantly different from 1900 K (one-way ANOVA, $p < 0.0001$). The data are presented as the mean ± SD of at least three independent experiments. Scale bars=20 μm.

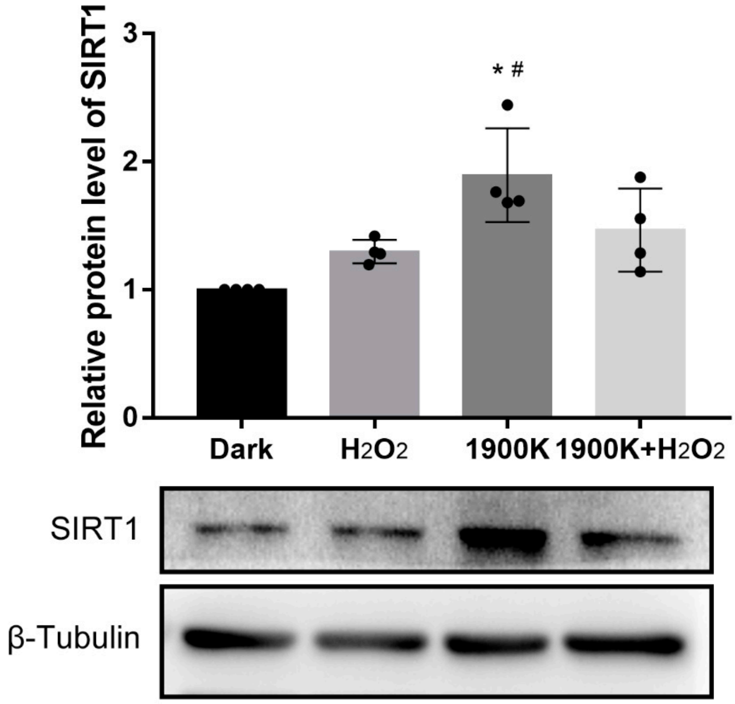

Figure S5. 1900 K LEDs pre-illumination was able to up-regulate the expression of Sirtuin 1 (SIRT1). The results show that the 1900 K LEDs can increase the level of SIRT1. H_2O_2 damage also has a tendency to increase SIRT1, with lower degree than that in the 1900 K group. The SIRT1 level of the 1900 K+H_2O_2 group is between the H_2O_2 group and the 1900 K group. The data are presented as the mean ± SD, n = 4. Asterisk (*): Significantly different from Dark (one-way ANOVA, 1900 K: p = 0.0013). Hash sign (#): Significantly different from H_2O_2 (one-way ANOVA, 1900 K: $p = 0.0240$).
